# Development and validation of the help-seeking intention scale in university students with hazardous and harmful consumption of alcohol

**DOI:** 10.3389/fpsyg.2023.1112810

**Published:** 2023-03-01

**Authors:** Daniela Romero Reyes, Juan Antonio Moriano León, José Luis Ybarra Sagarduy

**Affiliations:** ^1^International Doctoral School, Universidad Nacional de Educación a Distancia (UNED), Madrid, Spain; ^2^Department of Social and Organizational Psychology, Faculty of Psychology, Universidad Nacional de Educación a Distancia (UNED), Madrid, Spain; ^3^Academic Unit of Social Work and Sciences for Human Development, Universidad Autónoma de Tamaulipas, Ciudad Victoria, Tamaulipas, Mexico

**Keywords:** Theory of Planned Behavior, hazardous consumption of alcohol, harmful consumption of alcohol, university students, help-seeking

## Abstract

**Introduction:**

The Theory of Planned Behavior (TPB) has been proposed as suitable to study help-seeking intentions. This paper aims to develop the IH-RHAC scale (Help-seeking intention in young adults with hazardous and harmful alcohol consumption) with the TPB. The objectives of the study were: (a) to analyze the structure, reliability, and validity of the instrument, (b) to identify whether attitude, subjective norm, self-efficacy, and past help-seeking would predict help-seeking intention, and (c) to assess concurrent validity.

**Methods:**

From a total of 2,011 students who responded to the surveys, the sample was made up of 263 university students aged 18 to 29 with hazardous and harmful alcohol consumption practices, who responded to an online questionnaire including the AUDIT, IH-RHAC, and a scale of barriers and resources for alcohol consumption. Partial least squares structural equations (PLS-SEM) were used to test the hypotheses about reliability, validity of the scales, and prediction of the constructs: attitude, subjective norms, self-efficacy, and help-seeking in the past about intention. Pearson’s correlations were used to obtain evidence of concurrent validity.

**Results:**

The results displayed favorable psychometric characteristics. The internal measurement model showed that attitude, self-efficacy, and prior help-seeking predicted a 27% help-seeking variance. Subjective norm did not predict intention.

**Discussion:**

It has been concluded that this is an instrument with psychometric support that can contribute to designing and/or evaluating interventions that promote the students’ search for help.

## Introduction

Hazardous and harmful consumption of alcohol in university students is a public health problem (Davoren et al., 2016; Charles et al., 2021) that can have serious consequences in the lives of young adults (Krieger et al., 2018). A systematic review reported a median prevalence of harmful alcohol use in Latin American students of 26.2 (Rangel et al., 2017). In Mexico, young adults between the ages of 18 and 29 have the highest per capita alcohol consumption (7.6 liters) (Villatoro-Velázquez et al., 2017). In another study carried out with 19,815 Mexican university students, the prevalence of alcohol consumption was 55.5%. These young adults showed a greater probability of consuming illegal drugs (Gogeascoechea-Trejo et al., 2021).

Hazardous alcohol consumption expresses a pattern that leads to the risk of harmful physical, mental, and/or social consequences for the user (Babor and Higgins-Biddle, 2001). The International Classification of Diseases (ICD-11) states that a pattern of harmful use of alcohol can be continuous or episodic, causing damage to the mental and physical health of that person and/or other people. This type of classification seeks to help identify the negative impact of substance use early (Bobes et al., 2019).

Hazardous and harmful consumption of alcohol can be addressed through brief interventions aimed at reducing harmful consumption (Babor et al., 2001); however, in the university context, few young adults seek help (Codd and Cohen, 2003; Cellucci et al., 2006; Caldeira et al., 2009; Lowinger, 2012). Given this situation, carrying out research on the psychosocial factors that predict help-seeking is highly relevant.

Seeking help for health issues is a planned behavior. Furthermore, the attempt to solve the problem involves interacting with a healthcare professional (Cornally and McCarthy, 2011). According to Belló et al. (2008) help-seeking is the request for assistance from a person or institution to discuss alcohol consumption problems. Early help-seeking leads to early exposure to healthcare services that can alter the progression of use and prevent unfavorable consequences (Bhochhibhoya et al., 2015; Mota et al., 2019).

Instruments have been developed from various approaches to identify variables related to seeking help for alcohol consumption in university students. Some of them have addressed the stigma associated with seeking psychological help (Cellucci et al., 2006; Vogel et al., 2006), willingness to seek help (Lowinger, 2012), the probability of participating in treatment options (Buscemi et al., 2010), and the barriers to and resources for seeking help (Salazar et al., 2020).

The search for help for alcohol consumption in university students has been linked to numerous variables, however, these links have not been studied under a unified theoretical model (Salazar et al., 2020). A psychological aspect of the help-seeking process is intention (Tomczyk et al., 2020). For White et al. (2018), the help-seeking intention can be defined as a plan that implies an effort to express a problem in an attempt to get support to reduce one’s discomfort. The authors recognize that the Theory of Planned Behavior (TPB) is a complete model that covers the elements mentioned in the definition of the construct, so it is useful to study the help-seeking intention of young adults with hazardous and harmful alcohol consumption.

Moreover, the TPB is strongly supported by empirical evidence (Armitage and Conner, 2001; Cooke and French, 2008). It is highly accurate in predicting intentions of behavior through: attitude, subjective norm, and perceived behavioral control. The attitude refers the degree to which a person has a favorable or unfavorable evaluation of a behavior, and its antecedent is the behavioral beliefs that are beliefs about the possible consequences and experiences related to the behavior; the subjective norm indicates the perceived social pressure of important referents to perform or not perform a certain behavior, and it is preceded by normative beliefs that are beliefs about the normative expectations of significant other people; perceived behavioral control is described as the perceived ease or difficulty of performing a behavior and its antecedent are the control beliefs that are beliefs about the presence of factors that facilitate or impede the execution of the behavior (Ajzen, 1991).

The TPB is a reformulation of the Theory of Reasoned Action (Ajzen and Fishbein, 1980), and it includes perceived behavioral control, which is like self-efficacy and can be operationalized as such according to the needs of the study (Ajzen, 2002b) since that both refer to the perceived ability to perform a specific behavior (Bandura, 1982). The TPB proposes that the more favorable the attitude and the subjective norm and the greater the perceived behavioral control, the stronger the intention to perform the behavior, (Ajzen, 1991). In this way we can hypothesize that the intention to seek help would be preceded by attitudes, subjective norms, and perceived behavioral control (operationalized as self-efficacy). According to this theory, the intention represents the immediate antecedent of the behavior (Ajzen, 2002a), in this case the help-seeking behavior. Since the TPB was originally formulated, studies have been carried out to predict treatment seeking or retention in substance use disorder patients (Kleinman et al., 2002; Kelly et al., 2011, 2016; Vederhus et al., 2015; Zemore et al., 2021). Codd and Cohen (2003) worked from the Theory of Reasoned Action with university students, finding that the attitude and the subjective norms predicted a 12% variance in help-seeking intentions. However, they recommended using the TPB by integrating perceived behavioral control, a variable that could have a greater influence on help-seeking intention.

According to the literature, one predictor of treatment seeking in alcoholism is prior help seeking (Finney and Moos, 1995). Freyer et al. (2007) found that people who sought help in the past were 4.6 times more likely to attend self-help groups, 7.6 times more likely to go through detoxification, and 8.7 times more likely to be involved in outpatient treatment. Although their study covered a broad spectrum of users, the authors recommended conducting research on at-risk or harmful users to design timely early interventions. Based on the TPB, a measure of past behavior can be included to improve prediction of future actions (Ajzen, 2002c; Hagger et al., 2018).

**FIGURE 1:**
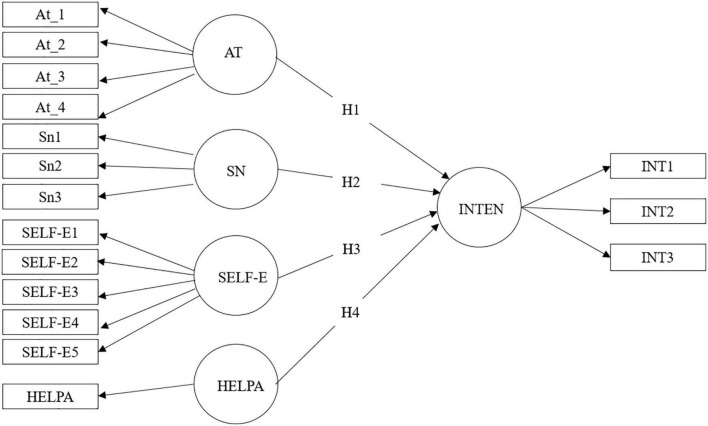
Theoretical model and hypothesis. At_1 = attitude 1 [(item 1 × item 5)/5], At_2 = attitude 2 [(item 2 × item 6)/5], At_3 = attitude 3 [(item 3 × item 7)/5], At_4 = attitude 4 [(item4 × item8)/5]; SN1 = subjective norm 1 [(item 1 × item 4)/5], SN2 = subjective norm 2 [(item 2 × item 5)/5], SN3 = subjective norm 3 [(item 3 × item 6)/5]; AT, attitude; SN, subjective norm; SELF-E, self-efficacy; HELPA, help-seeking in the past; INT, intention.

The TPB offers a convenient conceptual framework: its constructs shed light on specific behaviors in particular contexts (Ajzen, 1991). Therefore, this study aims to develop a psychometric scale called IH-RHAC (Help-seeking intention in young adults with hazardous and harmful alcohol consumption) based on the TPB that assesses attitudes, subjective norms, perceived behavioral control (assessed through self-efficacy), and the help-seeking intention of young adults with hazardous alcohol consumption. The objectives of this study are (a) to analyze the structure, reliability, and validity of the instrument’s scales and (b) to identify if attitude, subjective norms, and self-efficacy will predict help-seeking intention. To do so, the work was guided by the following hypotheses (see Figure 1).

Hypothesis 1. Attitude will be positively related to help-seeking intentions.

Hypothesis 2. Subjective norm will be positively related to help-seeking intention.

Hypothesis 3. Self-efficacy will be positively related to help-seeking intention.

Hypothesis 4. Past help-seeking will be positively related to help-seeking intention.

## Materials and methods

### Design

The aim of this instrumental study is to develop a scale to assess help-seeking and emphasis its psychometric properties (Ato et al., 2013).

### Sample

From a total of 2,011 students who responded to the surveys, we worked with a convenience sample of 263 students from two public universities in northeastern Mexico: 135 men and 128 women with an average age of 20.89 (SD = 2.24). The inclusion criteria were: (a) being 18 to 29 years old, (b) being a Mexican university student, (c) voluntary participation, (d) obtaining a score equal to or greater than five points in the Alcohol Use Disorders Identification Test (AUDIT, Saunders et al., 1993). The exclusion criteria were: (a) young adults who did not agree to participate in the study and (b) young adults with an AUDIT score below 5. Most of the participants (95.8%) reported being single, 39.2% reported studying a degree in the humanities and behavioral sciences, 85.2% indicated they lived with their parents, 48.7% mentioned they work in addition to studying. The median family monthly income was $424.90 (USD).

### Procedure

The corresponding permission was requested from the university authorities who in the academic committee approved the study. From 8 February 2021 to September 27 of that same year data was collected electronically by sharing the instruments on social media such as WhatsApp. A total of 2,011 students answered the surveys, of which 184 (9.14%) did not agree to participate in the study. Of the remaining 1,827 participants, only 318 met the hazardous and harmful consumption of alcohol criteria (AUDIT ≥ 5), of which 41 (12.89%) were cases with missing data, leaving a base of 277 complete cases. These were adjusted to the age criterion (18 to 29 years old), resulting in 263 complete cases suitable for statistical analysis.

### Instruments

#### Sociodemographic data sheet

The data sheet was built *ad hoc* by the researchers to obtain sociodemographic information for a detailed description of the sample.

#### Alcohol Use Disorders Identification Test (AUDIT)

This instrument is for international use and is validated in Mexico (Medina-Mora et al., 1998). This test identifies hazardous, harmful consumption and possible alcohol dependence through a 10-item questionnaire (Saunders et al., 1993). Each question has a series of options that are scored from 0 to 4. Scores equal to or greater than 8 indicate harmful consumption. A more rigorous interpretation can be made when analyzing the scored answers: a score equal to or greater than 1 in items 2 and 3 indicates hazardous consumption and a score above 0 in items 4–6 suggests the beginning of dependency (Babor et al., 2001). In this study, a score of 5 or more was considered to identify young adults with hazardous and harmful alcohol consumption. In research with students, this instrument has shown adequate internal consistency of up to 0.92 (Moral et al., 2017).

#### Help-seeking intention in young adults with hazardous and harmful alcohol consumption questionnaire (IH-RHAC)

The IH-RHAC measure is based on the TPB (Ajzen, 1991). The instrument was elaborated following the conceptual and methodological guidelines of Ajzen (2002a) for the construction of TPB questionnaires. The author establishes that measures based on beliefs can be designed, which contribute to explain the behavior not only to predict it. Theoretically the outstanding beliefs will be the determinants of a person’s intentions and actions: behavioral beliefs (precede attitude), normative beliefs (precede the subjective norm), and control beliefs (integrate perceived behavioral control) (Ajzen, 1991). For the creation of measures based on beliefs, Ajzen (2002a) recommends carrying out a pilot study to identify the salient beliefs of a sample of the target population; not doing so would imply the risk of arbitrarily including beliefs in the instruments that are not necessarily they would represent the beliefs of that population and compromise the quality of the scales (Ajzen, 1991).

Therefore, in a previous study, the behavioral, normative, and control beliefs underlying the help-seeking intention of college students with hazardous and harmful alcohol use were identified Following Ajzen’s (2002a) proposal, respondents from the previous study were given a description of the help-seeking behavior, followed by a series of open-ended questions about the advantages and disadvantages they considered about help-seeking (to obtain the behavioral beliefs), about the identity of the relevant referent individuals or groups that could approve or disapprove of their help-seeking (in this way the normative beliefs were obtained) and finally the factors or circumstances that would facilitate or hinder their behavior help-seeking (thus obtaining control beliefs).

Continuing with the procedure, those beliefs that were more frequent or that were significantly related to the intention to seek help were used to elaborate the items of the subscales of attitude, subjective norm, and perceived behavioral control. In this way the outstanding beliefs of the university students provided the basis for the formation of the preliminary questionnaire. Behavioral and normative beliefs were measured using the expectation-value model (Ajzen, 1991, 2002a) where the strength of the items that measure behavioral beliefs are combined in a multiplicative manner with the evaluation of the results, which generates a single attitude score. The strength of normative beliefs was multiplied by the motivation to comply resulting in the subjective norm. Perceived behavioral control was operationalized as self-efficacy. Then a pilot study was carried out where the preliminary scale was applied to 27 university students. The results of the piloting allowed us to identify the need to adjust the response options of the subjective norm scale, changing “strongly disagree-strongly agree” to “not at all agree–totally agree” to help participants understand the options better.

The final instrument aims to measure help-seeking intention and attitude, subjective norm, and perceived behavioral control (operationalized as self-efficacy) based on the beliefs underlying these constructs: behavioral beliefs, normative beliefs, respectively, including the variable seeking help in the past. The scale is composed of five sections: (a) attitude, (b) subjective norm, (c) self-efficacy, (d) help-seeking intentions, and (e) past help-seeking. Responses were scored on a Likert-type scale from 1 to 5 (see Supplementary material and Table 1).

**TABLE 1:** Dimensions and items of the IH-RHAC scale.

Dimensions	Items	Contents
Attitude		Section A. Indicate to what extent you consider that seeking help to solve problems with alcohol consumption would help you to:
	1	Learn to regulate alcohol consumption
	2	Improve my quality of life
	3	Stop drinking alcohol
	4	Make better decisions
		Now please indicate to what extent the following aspects are important to you in your life:
	5	Learn to regulate alcohol consumption
	6	Improve my quality of life
	7	Stop drinking alcohol
	8	Make better decisions
Subjective norm		Section B. Now please think about the people who are closest to you, to what degree would they agree if you sought help for your alcohol consumption?
	1	My parents and siblings
	2	My close friends
	3	My partner
		And how do you rate the opinion of these people in relation to seeking help for your alcohol consumption? I consider it…
	4	My parents and siblings
	5	My close friends
	6	My partner
Self-efficacy		Section C. To what degree do you think you would be able to perform each of the following behaviors?
	1	Obtain information about centers specialized in treating alcohol consumption
	2	Attend centers specialized in treating alcohol consumption
	3	Participate in an online program to treat alcohol consumption
	4	Contact a healthcare professional (psychologist, therapist) to treat alcohol consumption
	5	Seek support from my family and/or friends to help me with my alcohol use problem.
Intention		Section D.
	1	Do you intend to seek help for your alcohol consumption during the next month?
	2	Do you plan to seek help for your alcohol consumption?
	3	If you had the opportunity, would you want to seek help for your alcohol consumption?
		Section E.
Help-seeking in the past	1	Have you sought help in the past for your alcohol consumption?

(a) Attitude was assessed by means of eight items on beliefs about the consequences of seeking help and their assessment. This subscale had two dimensions: the four items of the first block measured the strength of the behavioral belief, for example: “Indicate to what extent you consider that seeking help to solve problems with alcohol consumption would help you learn to regulate consumption” with a Likert-type response scale from 1 (not at all) to 5 (a lot). The second dimension consisted of the evaluation of the results of these beliefs through four items, for example: “Now please indicate to what extent the following aspects are important to you in your life: learning to regulate consumption.” They were measured with a Likert-type response scale from 1 (not important at all) to 5 (very important). In this way, two people can have the same strength in a belief–“learn to regulate consumption”–, but they will be able to assess its result in a different way. For one it may be very important to learn to regulate consumption and for another it may not. The two dimensions were combined multiplicatively to obtain a single score, as can be seen in the following formula:

(1) $$Attitude=\left(\sum_{1}^{n}Attitude_{n}\right)/\left(n.items\right)=$$

[(item1×item5)/5+(item2×item6)/5+

(item3×item7)/5+(item4×item8)/5]/4

(b) The subjective norms were composed of two dimensions. The first was the strength of normative beliefs regarding the expectations of people close to the respondent: parents, siblings, friends, or partners. It consisted of three items, for example: “Please now think about the people who are closest to you. To what degree would they agree if you sought help for your alcohol consumption?” measured on a five point Likert type scale: 1 (do not agree) to 5 (totally agree). The second dimension was the motivation to meet normative expectations, made up of three items, for example: “How much do you value the opinion of these people in relation to seeking help for your alcohol consumption?” This was also evaluated with a five point Likert scale, where 1 represented not at all important and 5 very important. These dimensions were combined multiplicatively to obtain a single score, as shown in the following formula:

(2) $$Subjetive\_norm=\left(\sum_{1}^{n}SN_{N}\right)/\left(n.items\right)=$$

[(item1×item4)/5+(item2×item5)/5+

(item3×item6)/5]/3

(c) Auto-efficacy for help-seeking was composed of five items which sought to find out the degree in which young adults felt capable of carrying out certain behavioral tasks, like “obtaining information on treatment centers specializing in alcohol abuse.” For the evaluation we used a five point Likert scale where 1 represented incapable and 5 fully capable.
(d) Help-Seeking intention was composed of three items with the aim of identifying help-seeking intentions for alcohol consumption, like, for example, “If you had the opportunity, would you want to seek help for your alcohol consumption?” Its evaluation was measured using a five point Likert scale, where 1 meant definitely not and 5 -definitely.
(e) Help-Seeking in the past was evaluated by means of the question “Have you sought help in the past for your alcohol consumption?” with a dichotomous response of “yes” or “no.”

#### Barriers and resources for help-seeking in changing alcohol consumption by university students

The instrument was used to measure concurrent validity. This Mexican-designed test evaluates the barriers and environmental resources related to help-seeking in university students with excessive alcohol consumption (Salazar et al., 2020). It consists of two scales. The first is barriers, composed of nine items distributed into three factors: standard of morality, lack of social support from parents/social stigma, and lack of social support from peers. The second is scale of resources, composed of 12 items distributed in four factors: health systems and social support, negative consequences, parent/friend rejection, and parent/partner rejection. In young Mexican university students, the instrument obtained Cronbach’s Alpha of 0.86 for the barriers scale and 0.84 for the resources scale.

### Data analysis

The descriptive analysis of the data was carried out using SPSS version 21 to obtain the means, standard deviations, correlations, asymmetry, and kurtosis. Robust methods were used due to the non-normality of the distribution of the variables (Hair et al., 2019). The missing data were analyzed with Little’s MCAR test, which showed that the missing data were completely random (X2 = 394.358, gl. 521, *p* > 0.05). Therefore, the listwise deletion method was used, not including the records with missing data (Bentler, 2006). Next, partial least squares structural equations (PLS-SEM) were used to test hypotheses 1–4 raised in the study. PLS-SEM is a non-parametric technique for analyzing pathway models that are based on compounds. This method is recommended when the analysis is attempting to test the prediction of a theoretical framework, when the structural model is complex, i.e., composed of a considerable number of constructs and/or model relations, or when the route model is made up of one or more constructs formatively measured. It is also a robust method that offers solutions to small or large sample sizes and with abnormal distributions (Hair et al., 2019). The SmartPLS version 3 software was used.

The PLS-SEM analyzes are carried out in two phases. First, the external measurement model is evaluated where reliability is obtained through Cronbach’s Alpha, internal and convergent validity through the average variance extracted (AVE), and discriminant validity with the Fornell-Larcker criterion and the ratio HTML. The second phase consisted of evaluating the internal model, where the proposed hypotheses are tested (Martínez and Fierro, 2018; Hair et al., 2019). To obtain concurrent validity of the IH-RHAC scales with the barriers and resources scales, Pearson’s correlations were used through SPSS.

### Transparency and openness

We report how the sample was formed, data exclusions, all study measures, and follow the Journal Article Reporting Standards (JARS) recommendations (Appelbaum et al., 2018; Kazak, 2018). Data, analysis code, and research materials are available to applicants by writing an email to the author. Data were analyzed using SPSS version 21 and SmartPSL version 3. The design of this study and its analysis were not registered previously.

### Ethical considerations

The protocol was reviewed and accepted by an academic committee made up of the directors, research secretaries, and academic secretaries of the universities who authorized the instruments to be disseminated among the young adults. The participants read an informed consent beforehand in which the purpose of the study was explained to them, emphasis was placed on their freedom to stop or abandon the study at any time during the application without any consequence, and they were informed that the data collected would only be used within the context of the research.

## Results

### External measurement model

The measurement model shows the relationships between the latent variables and their indicators. The external measurement model consisted of the constructs of attitude, subjective norms, self-efficacy, past help-seeking, and intention. The individual reliability of each indicator was obtained by analyzing the loads with their latent variable. The relationships of all the indicators with their variables showed loads greater than 0.86, representing strong loads, since standardized external loads (λ) greater than 70 are considered adequate (Carmines and Zeller, 1979). Internal consistency using Cronbach’s Alpha was favorable for the latent variables: attitude (0.93), subjective norms (0.92), self-efficacy (0.95), intention (0.90). The average variance extracted (AVE) shows evidence of internal and convergent validity: the AVE must be = or >0.50 and indicates the variance that each construct acquires from its indicators in relation to the variance caused by the measurement error. The constructs obtained an adequate AVE of 0.83 for attitude, 0.87 for subjective norms, 0.87 for self-efficacy, 1.00 for help-seeking in the past, and 0.83 for intention (see Table 2).

**TABLE 2:** Factor loadings and reliability.

Latent variable	Item	λ	AVE	Composite reliability	Cronbach’s Alpha
Attitude	At_1	0.92	0.83	0.95	0.93
	At_2	0.88			
	At_3	0.93			
	At_4	0.90			
Subjective norms	SN1	0.93	0.87	0.95	0.92
	SN2	0.94			
	SN3	0.92			
Self-efficacy	SELF-E1	0.93	0.87	0.96	0.95
	SELF-E3	0.94			
	SELF-E4	0.94			
	SELF-E5	0.92			
Help-seeking	HELPA	1	1	1	1
Intention	INT1	0.94	0.83	0.93	0.92
	INT2	0.93			
	INT3	0.86			

λ = factor loadings, AVE = average variance extracted; At_1 = attitude 1 [(item 1 × item 5)/5], At_2 = attitude 2 [(item 2 × item 6)/5], At_3 = attitude 3 [(item 3 × item 7)/5], At_4 = attitude 4 [(item 4 × item 8)/5]; SN1 = subjective norm 1 [(item 1 × item 4)/5], SN2 = subjective norm 2 [(item 2 × item 5)/5], SN3 = subjective norm 3 [(item 3 × item 6)/5]; SELF-E, self-efficacy; HELPA, help-seeking in the past; INT, intention.

The Fornell-Larcker criterion was used to obtain discriminant validity, which considers that the square root of the AVE of an item should be greater than its correlation with other items. In this instrument, the loads of the indicators in their corresponding construct were greater than the cross loads with other constructs and the square root of the AVE of each construct is greater than the correlations with the others, as can be seen in Table 3.

**TABLE 3:** Discriminant validity (Fornell-Larcker criterion).

Latent constructs	Self-efficacy	Attitude	HELPA	Intention	Sn
Self-efficacy	(0.93)				
Attitude	0.43	(0.91)			
HELPA	0.05	0.01	(1.00)		
Intention	0.34	0.34	0.34	(0.91)	
Subjective norms	0.52	0.53	0.01	0.28	(0.93)

The square root of the AVE is shown diagonally in parentheses.

HELPA, help-seeking in the past, Sn, subjective norm.

According to Henseler et al. (2015), the HTMT ratio better identifies the lack of validity. There are discriminant validity problems when the HTMT value is above the 0.90 threshold. In this study, all the constructs obtained a HTMT value below 0.85, which is an even more conservative limit, thus showing adequate discriminant validity (see Table 4).

**TABLE 4:** The HTMT ratio with SmartPLS algorithm.

Construct	Self-efficacy	Attitude	HELPA	Intention
Self-efficacy				
Attitude	0.44			
HELPA	0.05	0.02		
Intention	0.37	0.35	0.36	
Subjective norm	0.55	0.57	0.03	0.30

HELPA, help-seeking in the past.

Finally, the maximum values of the variance inflation factor (VIF) were 8.18 for indicator 2 of self-efficacy, so the decision was made to eliminate it from the instrument, after which 5.60 was obtained for indicator 4 of self-efficacy, and 5.26 for indicator 3 of self-efficacy. All the others remained under five so it was concluded that there were no multicollinearity problems.

### Descriptive results and correlations between variables

Descriptive statistics related to means and standard deviations are shown in Table 5. Pearson’s correlation matrix reveals provisional support for the hypotheses. Attitude, subjective norm, self-efficacy, and help-seeking in the past were positively associated with help-seeking intention (*r* = 0.32, *p* < 0.05; *r* = 0.28, *p* < 0.05; *r* = 0.35, *p* < 0.05, *r* = 0.33, *p* < 0.05).

**TABLE 5:** Descriptive and Pearson’s correlations between constructs.

Constructs	Mean	SD	1	2	3	4	5	6
1. Age	20.89	2.24						
2. Sex	1.51	0.50	0.00					
3. Attitude	2.11	1.39	0.00	0.03				
4. Subjective norm	2.64	1.57	0.01	0.12*	0.53**			
5. Self-efficacy	3.07	1.42	0.00	0.00	0.41**	0.52**		
6. HELPA	0.09	0.28	0.01	0.05	0.00	0.01	0.05	
7. Intention	1.89	1.19	0.10	0.01	0.32**	0.28**	0.35**	0.33**

*p < 0.05, **p < 0.01.

SD, standard deviation; HELPA, help-seeking in the past.

### Internal measurement model: Hypothesis test

**FIGURE 2:**
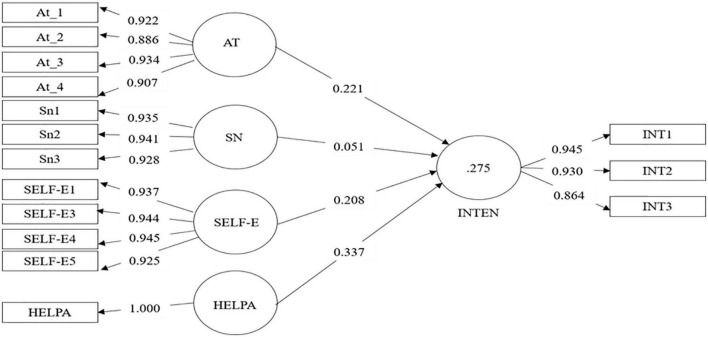
Path model estimation. At_1 = attitude 1 [(item 1 × item 5)/5], At_2 = attitude 2 [(item 2 × item 6)/5], At_3 = attitude 3 [(item 3 × item 7)/5], At_4 = attitude 4 [(item 4 × item 8)/5]; SN1 = subjective norm 1 [(item 1 × item 4)/5], SN2 = subjective norm 2 [(item 2 × item 5)/5], SN3 = subjective norm 3 [(item 3 × item 6)/5]; AT, attitude; SN, subjective norm; SELF-E, self-efficacy; HELPA, help-seeking in the past; INT, intention.

The relationships between all the variables proposed in the model can be seen in Figure 2. Attitude is significantly related to intention (ß = 0.22, *p* < 0.001), admitting H1. Subjective norms were not significantly related to intention so H2 was rejected, self-efficacy was significantly associated with intention (ß = 0.20, *p* < 0.001) supporting H3, past help-seeking was significantly associated with intention (ß = 0.32, *p* < 0.001) affirming H4. This general model explained 27% of the variance in help-seeking intention (see Table 6).

**TABLE 6:** Path coefficients (standardized regression coefficients).

Hypothetical relationships	Path coefficients (Standardized ß)	Student’s *t*-statistic	*p*-value
At →IN	0.22	3.23	0.001
Sn →IN	0.05	0.77	0.436
Self-e →IN	0.20	3.75	0.001
HELPA →IN	0.32	4.58	0.001

At, attitude; Sn, subjective norm; Self-e, self-efficacy; HELPA, help-seeking in the past; IN, intention.

The predictive relevance of the PLS route model was evaluated with the Stone-Geisser calculations, obtaining the Q2 (Hair et al., 2019). The Blindfolding procedure (*D* = 7) and the redundancy approach with cross validation were applied and it was found that self-efficacy presented Q2 = 0.77, being the highest value, followed by attitude and subjective norm with Q2 = 0.69, followed by intention with Q2 = 0.63. The results show that the model has predictive relevance for these constructs as they are values greater than 0.

Finally, effect sizes were evaluated using f2. The effect sizes of attitude and self-efficacy on intention were small (f2 = 0.04) and the effect size of past help-seeking on intention was medium (f2 = 0.14) (see Table 7).

**TABLE 7:** *f*^2^ effect sizes.

Exogenous constructs	Endogenous construct intention	
	Path coefficients	*f*^2^ effects
Attitude	0.22	0.04
Self-efficacy	0.20	0.04
HELPA	0.32	0.14

HELPA, help-seeking in the past.

### Evidence of concurrent validity of IH-RHAC with barrier and resource scales

The Pearson correlation coefficient was used to obtain concurrent validity. Attitude and intention showed significant correlation with help-seeking barriers; attitude, subjective norm, and self-efficacy showed significant correlation with help-seeking resources (see Table 8).

**TABLE 8:** Pearson correlations of IH-RHAC scales with barrier and resource scales.

	Attitude	Sn	Self-efficacy	HELPA	Intention
Barriers	0.15*	0.09	0.12	0.11	0.26**
Resources	0.28**	0.40**	0.41**	-0.04	0.11

*p < 0.05, **p < 0.01.

Sn, subjective norm; HELPA, help-seeking in the past.

When correlating the IH-RHAC scales with the barriers to alcohol consumption subscale, the attitude correlated with the absence of social support/stigma, the search for help in the past with morality standards, and the intention with the morality standards, absence of social support/stigma, and lack of family support (see Table 9).

**TABLE 9:** Correlations of Pearson IH-RHAC scales with barrier subscales.

	Morality	No social support and stigma	No family support
Attitude	0.10	0.15*	0.11
Subjective norm	0.06	0.09	0.08
Self-efficacy	0.08	0.11	0.11
HELPA	0.14*	0.06	0.09
Intention	0.21**	0.23**	0.27**

*p < 0.01, **p < 0.05.

HELPA, help-seeking in the past.

Attitude, subjective norms, and self-efficacy correlate with the four resource subscales. Attitude and self-efficacy have greater correlations with access to services, subjective norms with rejection from parents/friends (see Table 10).

**TABLE 10:** Correlations of Pearson IH-RHAC scales with resource subscales.

	Services	Conse- quences	Parent/Friends rejection	Parent/Partner rejection
Attitude	0.30**	0.23**	0.24**	0.22**
Sn	0.37**	0.35**	0.39**	0.35**
Self-efficacy	0.42**	0.31**	0.41**	0.36**
HELPA	-0.02	-0.07	-0.03	-0.04
Intention	0.14*	0.05	0.13*	0.08

*p < 0.01, **p < 0.05.

Sn, subjective norm; HELPA, help-seeking in the past.

## Discussion

The objective of this study was to develop a psychometric scale from TPB that would assess attitudes, subjective norms, self-efficacy, past help-seeking, and help-seeking intentions of young adults with hazardous and harmful alcohol consumption. The specific objectives were to analyze the structure, reliability, and validity of the instrument’s scales and to identify whether attitude, subjective norms, self-efficacy, and prior help-seeking would predict help-seeking intentions. According to the results, the IH-RHAC instrument presented favorable psychometric characteristics. The reliability of each subscale was satisfactory.

## Theoretical and research implications

Regarding the proposed predictive model, it was found that the attitude predicted help-seeking intentions, which is consistent with other studies that have addressed the search for treatment using TPB in substance dependent people (Kelly et al., 2011, 2016; Vederhus et al., 2015) as well as what was found in university students with mental health problems or harmful alcohol consumption (Codd and Cohen, 2003; Lee and Shin, 2022). In this study, attitude was not the strongest predictor of intention in contrast to others for which it was (Schomerus et al., 2009; Aldalaykeh et al., 2019). Subjective norms did not predict help-seeking intentions, similar to the findings in Kelly et al. (2011) and Bohon et al. (2016). There is evidence that this construct has a weaker relationship with the intention to attend screening (Cooke and French, 2008) or in university students who seek help at mental health services (Aldalaykeh et al., 2019). Attitude, subjective norms, and perceived behavioral control are expected to vary in predicting intentions according to different behaviors and situations (Ajzen, 1991). In university students with hazardous and harmful consumption of alcohol, perceived social pressure was not decisive for help-seeking intention.

Following Bohon et al. (2016), young adults may consider that their role models hold beliefs like theirs about seeking help, which contributes to understanding that subjective norms have not been predictors of intention. These results on perceived social pressure are also similar to those reported by Salazar et al. (2020) who indicated that young adults with a hazardous and harmful consumption of alcohol were surrounded by friends and relatives who drank excessively and had no social support from parents and friends in seeking help, which shows the influence of the descriptive norms that could be incorporated into the model as proposed by Kelly et al. (2011).

Self-efficacy was significantly associated with help-seeking intention. This result supports what was found in different investigations on the intention to start treatment in patients with alcohol abuse (Kelly et al., 2011) or in university students who seek help from mental healthcare services (Bohon et al., 2016; Aldalaykeh et al., 2019; Lee and Shin, 2022). In this regard, Lee and Shin (2022) suggest studying the knowledge that young adults have about the location and use of the different assistance services, since this knowledge impacts perceived behavioral control and therefore increases the intention to receive help.

Past help-seeking was the strongest predictor of help-seeking intention. Ajzen (2002c) points out that past behavior is a good predictor of subsequent behavior when attitudes and intention are ambivalent and do not offer clear guidelines for action, which could be understood when addressing a particularly sensitive issue due to stigma and social discrimination associated with alcohol use problems (Loureiro, 2013). Under these conditions including this measure in the prediction models was valuable.

The help-seeking construct can be difficult to measure. Wei et al.’s (2015) of the evaluation of knowledge, attitudes, and seeking help for mental health issues highlights the importance of validating the measurement instruments. In this sense, this scale has psychometric support, it was designed under an integrating model, it is concise and can be applied individually or collectively, in traditional or electronic format, it was created to evaluate the university population, it can be used by teachers, psychologists, and it can also be used to evaluate mental health literacy programs that promote help-seeking for alcohol-related problems.

This scale differs from others (Cellucci et al., 2006; Vogel et al., 2006; Caldeira et al., 2009; Lowinger, 2012; Salazar et al., 2020) in that it is based on a solid theoretical framework and is recommended for studying the help-seeking variable (White et al., 2018), in addition to adhering to Ajzen’s (2002a) by having carried out a pilot study to identify beliefs that supported the design of its items. The results found through the model can help shape effective interventions focused on changing attitudes and increasing self-efficacies that promote the help-seeking of students from support services on or off campus.

## Limitations and future lines of research

The limitations of this study were, first, not to evaluate the help-seeking behavior itself, followed by the possible effect of social desirability as it is a self-reporting instrument, and convenience sampling limits results generalization. A future line of research might be to evaluate this instrument’s predictive validity. Following Lee and Shin (2022), researching young adults’ knowledge of the mental healthcare services available on or off campus to check if it has an impact on perceived behavioral control and therefore on the formation of intention and help-seeking behavior would be important.

## Conclusion

IH-RHAC presented adequate psychometric characteristics, evidence of its construct, convergent, discriminant, and concurrent validity was obtained, and adequate reliability indices were attained. Subsequent studies should obtain evidence of the predictive validity of the instrument by evaluating help-seeking behavior. In this paper, the advantages of using the non-parametric PLS-SEM technique to test the prediction of TPB in help-seeking behavior were observed, a technique that allowed the researchers to acquire evidence of the instrument’s construct validity.

## Data availability statement

The raw data supporting the conclusions of this article will be made available by the authors, without undue reservation.

## Ethics statement

The studies involving human participants were reviewed and approved by Institutional Academic Committee of the Autonomous University of Tamaulipas. The patients/participants provided their written informed consent to participate in this study.

## Author contributions

DR: conceptualization, methodology, investigation, writing—original draft preparation, software, and formal analysis. JM: supervision, software, formal analysis, visualization, and writing—review and editing. JY: supervision and writing—review and editing. All authors contributed to the article and approved the submitted version.
