# Supplementary material for: Development and validation of the help-seeking intention scale in university students with hazardous and harmful consumption of alcohol
Source: Front Psychol. 2023 Mar 1;14:1112810. doi: 10.3389/fpsyg.2023.1112810 (PMC10014607; doi:10.3389/fpsyg.2023.1112810)
Supplement: Supplementary file 1 [file Data_Sheet_1.docx]

Supplementary Material

Development and validation of the Help-Seeking Intention Scale in university students with hazardous and harmful consumption of alcohol

**Scale IH-RHAC (English version)**

Section A. Indicate to what extent you consider that seeking help to solve problems with alcohol consumption would help you to:

1.- Learn to regulate alcohol consumption

2.- Improve my quality of life

3.- Stop drinking alcohol

4.- Make better decisions

Now please indicate to what extent the following aspects are important to you in your life:

5.- Learn to regulate alcohol consumption

6.- Improve my quality of life

7.- Stop drinking alcohol

8.- Make better decisions

Section B. Now please think about the people who are closest to you, to what degree would they agree if you sought help for your alcohol consumption?

1.- My parents and siblings

2.- My close friends

3.- My partner

And how do you rate the opinion of these people in relation to seeking help for your alcohol consumption? I consider it...

4.- My parents and siblings

5.- My close friends

6.- My partner

Section C. To what degree do you think you would be able to perform each of the following behaviors?

1.- Obtain information about centers specialized in treating alcohol consumption

2.- Attend centers specialized in treating alcohol consumption

3.- Participate in an online program to treat alcohol consumption

4.- Contact a health care professional (psychologist, therapist) to treat alcohol consumption

5. Seek support from my family and/or friends to help me with my alcohol use problem.

Section D.

1.- Do you intend to seek help for your alcohol consumption during the next month?

2.- Do you plan to seek help for your alcohol consumption?

3.- If you had the opportunity, would you want to seek help for your alcohol consumption?

Section E.

1.- Have you sought help in the past for your alcohol consumption? Yes___ No ___

**IBA-COPA (Spanish versión)**

Sección A. Señala hasta qué punto consideras que buscar ayuda para resolver problemas con el consumo de alcohol, te ayudaría a:

1. Aprender a regular el consumo de alcohol
2. Mejorar mi calidad de vida
3. Dejar de tomar alcohol
4. Tomar mejores decisiones

Ahora indica, por favor, hasta qué punto son importantes para ti en tu vida los siguientes aspectos:

1. Aprender a regular el consumo de alcohol
2. Mejorar mi calidad de vida
3. Dejar de tomar alcohol
4. Tomar mejores decisiones

Sección B. Por favor, ahora piensa en las personas que son más cercanas a ti, ¿en qué grado se mostrarían de acuerdo si buscaras ayuda por tu consumo de alcohol?

1. Mis padres y hermanos
2. Mis amigos/as cercanos/as
3. Mi novia(o) o pareja

Y ¿cómo valoras la opinión de estas personas en relación con la búsqueda de ayuda por tu consumo de alcohol?

1. Mis padres y hermanos
2. Mis amigos/as cercanos/as
3. Mi novia(o) o pareja

Sección C. ¿En qué grado crees que serías capaz de realizar cada una de las siguientes conductas?

1.- Obtener información sobre centros especializados en tratar el consumo de alcohol

2.- Asistir a centros especializados en tratar el consumo de alcohol

3.- Participar en un programa por internet para tratar el consumo de alcohol

4.- Contactar a un profesional de la salud (psicólogo, terapeuta) para tratar el consumo de alcohol

5.- Buscar apoyo de mi familia y/o amigos/as para que me ayuden con mi problema de consumo de alcohol.

Sección D.

1.- ¿Tienes intención de buscar ayuda por tu consumo de alcohol durante el próximo mes?

2.- ¿Tienes planeado buscar ayuda por tu consumo de alcohol?

3.- Si tuvieras la oportunidad ¿Querrías buscar ayuda por tu consumo de alcohol?

Sección E

1.- ¿Has buscado ayuda en el pasado por tu consumo de alcohol? Sí ___ No ___
